# Are the Associations between Life-style Related Factors and Plasma Total Homocysteine Concentration Different According to Polymorphism of 5,10-Methylenetetrahydrofolate Reductase Gene (C677T MTHFR)? - A Cross-sectional Study in a Japanese Rural Population -

**DOI:** 10.2188/jea.12.126

**Published:** 2007-11-30

**Authors:** Htay Lwin, Tetsuji Yokoyama, Chigusa Date, Nobuo Yoshiike, Yoshihiro Kokubo, Heizo Tanaka

**Affiliations:** 1 Department of Epidemiology, Medical Research Institute, Tokyo Medical and Dental University, 2-3-10, Kanda-surugadai, Chiyoda-ku, Tokyo 101-0062, Japan.; 2 Department of Public Health, Osaka City University Medical School, Osaka, Japan.; 3 Division of Health and Nutrition Monitoring, National Institute of Health and Nutrition, Tokyo, Japan.; 4 Department of Preventive Cardiology, National Cardiovascular Center, Osaka, Japan.; 5 National Institute of Health and Nutrition, Tokyo, Japan.

**Keywords:** homocysteine, MTHFR, serum folate and vitamin B_12_, smoking, Japanese

## Abstract

Mild hyperhomocysteinemia is one of the known strong risk factors for atherosclerotic diseases, and therefore it is important to clarify factors that could determine plasma total homocysteine (tHcy) level. A cross-sectional study with a random sample of 455 Japanese rural residents aged 40-69 years was conducted in 2000 to investigate the associations of plasma tHcy concentration with 5,10-methylenetetrahydrofolate reductase (MTHFR) gene and selected life-style related factors. The frequency of the mutant allele, Valine (V) allele, was 0.40 and the prevalence of VV genotype was 14.3 %. Plasma tHcy concentration in VV was significantly higher than those in two other genotypes. There were significant inverse associations of plasma tHcy with serum folate and serum vitamin B_12_ (P<0.001 for trend, respectively); both being stronger in VV than in other genotypes. The number of cigarettes smoked per day was positively associated with plasma tHcy concentration. A multivariate regression analysis revealed that serum folate, serum vitamin B_12_, and MTHFR genotype were independently associated with plasma tHcy. The inter-individual variance of plasma tHcy was more explained by serum folate and vitamin B_12_ than by MTHFR genotype. Higher intakes of folate, vitamin B_12_, and non-smoking may be important to prevent mild hyperhomocysteinemia and the eventual atherosclerotic diseases in this Japanese rural population.

## INTRODUCTION

Epidemiological evidence indicates that higher plasma concentration of total homocysteine (tHcy), a thio containing amino acid metabolized by either remethylation to metionine or transsulfuration to cysteine, is a risk factor for atherosclerotic diseases such as coronary, cerebral, and peripheral arterial diseases^1^^-^^3^^)^. To improve programs for the primary prevention of these atherosclerotic diseases, it is important to clarify factors, whether genetic or environmental, that could determine the levels of plasma tHcy. A common genetic factor, 5,10-Methylenetetrahydrofolate reductase (MTHFR) gene, and dietary factors such as folate, vitamin B_12_, and vitamin B_6_ have been suggested previously^1^^-^^5^^)^. Although severe hyperhomocysteinemia is rare, mild hyperhomocysteinemia occurs in approximately 5 % of general population due to these factors^1^^,^^2^^,^^5^^)^ and may be prevented by life-style modification.

In the present cross-sectional study conducted in a Japanese rural population, we analyzed the associations of plasma tHcy concentration with polymorphism of C677T MTHFR, serum concentrations of folate and vitamins B_12_ and B_6_, daily physical activity, smoking, and alcohol drinking to examine the effects of these genetic and environmental factors on the plasma tHcy concentration. Furthermore, we analyzed associations between plasma tHcy concentrations and the environmental factors in each of MTHFR genotypes separately because the metabolic pathways of homocysteine involve MTHFR and many cofactors (e.g. folate, vitamin B_12_, and vitamin B_6_) and, therefore, the strength of association between plasma tHcy concentration and environmental factors might be affected by the MTHFR genotype. Such knowledge about so-called the gene-environment interactions would be helpful to develop an optimal program of life-style modification to control mild hyperhomocysteinemia for individuals with different MTHFR genotypes.

## MATERIALS AND METHODS

### Study subjects

The participants were randomly selected from all residents aged 40-69 years in Shiso, a rural county located in the northwestern part of Hyogo Prefecture, Japan, in 2000. The present cross-sectional study was carried out as a part of a longitudinal study to continuously monitor the changes in life-styles and risk factors of cardiovascular diseases in this area during the last decade^6^^)^. The sample size was determined to detect a statistically significant difference of plasma tHcy levels according to MTHFR genotypes on the basis of some previous studies^1^^,^^7^^-^^11^^)^. All participants were assembled in local community halls, where 213 men and 242 women completed the following examinations.

The present study was approved by the ethics review committee of Medical Research Institute, Tokyo Medical and Dental University. All the subjects were taken for written informed consent.

### Assessment of life-style factors

Under a supervision of nurses or dietitians specifically trained for this study, each of the participants completed standardized questionnaires that included items about physical activity, smoking and alcohol drinking habits, and others. Smoking habit was classified into three groups as non-smokers (including ex-smokers), 1-19, and 20+ cigarettes/day. Daily alcohol drinking was also classified into three groups as 0-2.0, 2.1-4.0, and 4.1+ drinks/day, where one drink is approximately 12 grams of ethanol. Smoking and alcohol drinking were analyzed only in men because the prevalence of these was markedly higher in men than that in women. To evaluate the usual degree of physical activity, we used the intensity of physical activity as determined by a metabolic equivalents (METs) score, which was calculated as follows. Aside from sleeping time, the frequency and average duration of various types of labor and other activities on the job, including household activities, for every 2 months within the last 12 months were queried^6^^)^. One MET is as the resting metabolic rate, or approximately equivalent to 1 kcal/kg body weight per hour^12^^)^. In this report, we used the ‘active intensity index’ that was defined as the average METs during the time except sleeping. Body fat percentage was measured by the fitness analyzer, BFT-2000 (Kett Electric Laboratory, Japan), which was based on the near-infrared interactance technology. We measured serum concentrations of folate, vitamin B_12_, and vitamin B_6_ (pyridoxal 5′-phoshate), because their blood concentrations were known as biochemical indicators of dietary intake^13^^)^.

### Blood collection and measurements

Venous blood was drawn into EDTA-tube and serum tube after an overnight (12-h) fast. Then, the sample was immediately prepared at 4°C, and frozen and kept at - 20°C. The level of plasma tHcy was determined as total by high-performance liquid chromatography (HPLC) with fluorescence detection^14^^)^. Serum folate and serum vitamin B_12_ were measured by using Chemiluminescent analyzer ACS-180 (Chiron Corp, Califonia). Serum vitamin B_6_ (pyridoxal-5′-phosphate) levels were measured by using a HPLC equipped with a fluorescence detector.

### Genetic analysis

DNA was extracted from white blood cells with Puregene (Gentra Systems, Inc). Genotyping for MTHFR polymorphism was performed by polymerase chain reaction (PCR), with using the primers. The sequences of primers used in this study were the sense primer (5′-CAA AGG CCA CCC CGA AGC-3′) and anti-sense primer (5′-AGG ACG GTG CGG TGA GAG TG-3′). All PCRs to detect the MTHFR mutation were performed (amplified for 35 cycles consisting of denaturing at 94°C for 30 sec, annealing at 60°C for 60 sec and extension at 72°C for 60 sec, followed by a final extension step at 72°C for 10 min). Genotype was determined by digestion with Hinfl (Takara Co. Ltd), followed by 10 % polyacrylamide gel electrophoresis and ethidium bromide (1µg/ml) staining^7^^,^^8^^,^^15^^,^^16^^,^^17^^)^.

### Statistical analyses

Plasma tHcy, serum folate, serum vitamin B_12_, serum vitamin B_6_, and active intensity index were expressed as means ± SDs or logarithmic means ± SDs . Since the frequency distribution of plasma tHcy, the dependent variable in the present study, was skewed to the right side, the values were logarithmically transformed to follow an approximate normal distribution. Frequencies of alleles and genotypes of MTHFR were shown in percentage. Deviation of the genotype distribution from Hardy-Weinberg equilibrium was tested by *χ*^2^ analysis.

As for life-style factors, smoking and drinking were respectively classified into three groups as mentioned above. The values of physical activity, serum folate, serum vitamin B_12_, and serum vitamin B_6_ were logarithmically transformed for the same reason as plasma tHcy.

The associations of plasma tHcy with MTHFR genotype or selected life-style factors were examined by analysis of variance (ANOVA) followed by Tukey’s multiple comparison tests. Age- and sex-adjusted least square mean (LSM) of plasma tHcy in each of the combination between MTHFR genotypes and categories of life-style factors (categorized by tertiles for continuous variables) was calculated by analysis of covariance (ANCOVA). The strength of association between plasma tHcy and life-style factors was expressed as a standardized regression coefficient (std β) for each of the MTHFR genotypes, and difference of the std βs among the genotypes was tested by including the interaction term of life-style factor × genotype. Finally, the multiple regression analysis was performed to examine the independent contribution of MTHFR genotype and life-style factors to plasma tHcy, and the independent variables were sex, age, MTHFR genotypes, serum folate, serum vitamin B_12_ and interaction terms of serum folate × MTHFR genotypes which were significantly associated with plasma tHcy in the sex- and age-adjusted univariate analysis. All of the analyses were done with SAS statistical package (version 6.12, SAS Institute).

## RESULTS

### Characteristics of the study subjects

Table 1 shows the basic characteristics of the subjects participating in this study. Mean age of men and women was 49.0 ± 8.2 years and 49.7 ± 8.2 years, respectively. The mean level of plasma tHcy was significantly higher in men than in women. The frequency of VV genotype in men (16 %) was slightly higher than that in women (12.9 %) although not statistically significant. Serum folate was significantly higher in women than in men. The prevalence of male smokers (20+ cigarettes/day) was 45.4 % and that of male drinkers (4.1+ drinks/day) was 28.3 %.

**Table 1.** Basic characteristics of the subjects in Shiso, a Japanese rural county, in 2000.

	Men (n=213)	Women (n=242)
Age, yr	49.0 ± 8.2	49.7 ± 8.2
Plasma tHcy, µmol/l	13.3 ± 3.1	9.9 ± 2.5*
	(2.56 ± 0.21)†	(2.27 ± 0.21)†
MTHFR genotype‡		
AA	31.9%	36.3%
AV	52.1%	50.8%
VV	16.0%	12.9%
Serum folate, ng/ml	7.8 ± 3.5	10.3 ± 3.8*
	(1.96 ± 0.41)†	(2.26 ± 0.35)†
Serum vitamin B_12_, ng/ml	640.8 ± 296.6	726.5 ± 460.1
	(6.39 ± 0.36)†	(6.49 ± 0.41)†
Serum vitamin B_6_, ng/ml	24.3 ± 87.4	16.8 ± 21.0
	(2.60 ± 0.77)†	(2.48 ± 0.73)†
Smoking (cigarettes/day)		
0	43.9%	97.9%
1 - 19	10.7%	1.7%
20 +	45.4%	0.4%
Alcohol drinking (drinks/day )/¶		
0 - 2.0	46.2%	94.2%
2.1 - 4.0	25.5%	3.7%
4.1+	28.3%	2.1%
BMI (kg/m^2^)	23.2 ± 2.9	22.7 ± 3.1
Active intensity index, METs	2.05 ± 0.43	2.01 ± 0.32
	(0.70 ± 0.20)†	(0.69 ± 0.15)†

Values are means ± SDs or percentages.

* P< 0.05 for comparison between sexes.

† Means ± SDs of log-transformed values

‡ *χ*^2^ = 2.09, df = 1, P = 0.48 for comparison with Hardy-Weinberg equilibrium among all subjects.

¶ One drink is approximately 12 grams of ethanol.

### Plasma tHcy and C677T MTHFR genotypes

The frequency of mutant allele (V allele) of MTHFR was 0.40; the frequencies of three genotypes were AA 34.3%, AV 51.4%, and VV 14.3%. The distribution of the genotypes did not differ from the values expected for the Hardy-Weinberg equilibrium (χ^2^ = 2.09, df = 1, *P* = 0.48). Age- and sex-adjusted LSM of plasma tHcy level in MTHFR mutant genotype (VV) was significantly higher than those in two other genotypes (Table 2).

**Table 2.** Plasma total homocysteine level according to MTHFR genotype.

	MTHFR genotype			P*

	AA (n=156)	AV (n=234)	VV (n=65)	
Plasma tHcy, µmol/l	11.1 ± 0.21†	11.3 ± 0.18‡	13.3 ± 0.34	0.0001

Values are age- and sex-adjusted least square means ± SEs.

* P for homogeneity among the genotype groups.

† P< 0.05 for AA vs VV.

‡ P<0.05 for AV vs VV by Tukey’s multiple comparison.

### Plasma tHcy and life-style factors

Table 3 shows LSM of plasma tHcy (log and non log-transformed values) by tertile of selected life-style factors. Both serum folate and serum vitamin B_12_ were inversely correlated with plasma tHcy; smoking was positively correlated to plasma tHcy. There were no statistically significant associations of plasma tHcy with serum vitamin B_6_, alcohol drinking, BMI, and physical activity.

**Table 3.** Plasma total homocysteine and selected life-style factors.

	Plasma tHcy (log µmol/l)*	Plasma tHcy (µmol/l)‡
	
*Serum folate (log ng/ml)†*		
≤ 1.96	2.48 ± 0.02	12.5 ± 0.2
> 1.96 and ≤ 2.28	2.42 ± 0.02	11.6 ± 0.2
> 2.28	2.33 ± 0.02	10.5 ± 0.2
Std β ± SE	-0.32 ± 0.04	-0.33 ± 0.04
P for trend	0.0001	0.0001
*Serum Vitamin B_12_ (log ng/ml)†*		
≤ 6.27	2.46 ± 0.02	12.1 ± 0.2
> 6.27 and ≤ 6.55	2.40 ± 0.02	11.5 ± 0.2
> 6.55	2.36 ± 0.02	10.9 ± 0.2
Std β ± SE	-0.16 ± 0.04	-0.17 ± 0.04
P for trend	0.0001	0.0001
*Serum Vitamin B_6_ (log ng/ml)†*		
≤ 2.16	2.41 ± 0.02	11.5 ± 0.2
> 2.16 and ≤ 2.67	2.40 ± 0.02	11.5 ± 0.2
> 2.67	2.39 ± 0.02	11.3 ± 0.2
Std β ± SE	-0.01 ± 0.04	-0.005 ± 0.05
P for trend	0.77	0.91
*Smoking (cigarettes/day) (Men)*		
0	2.53 ± 0.02	12.7 ± 0.3
1∼19	2.54 ± 0.04	12.8 ± 0.6
≥ 20	2.60 ± 0.02	13.9 ± 0.3
β ± SE	0.12 ± 0.04¶	0.12 ± 0.04¶
P for trend	0.006	0.008
*Alcohol drinking (drinks/day) (Men)*		
≤ 2	2.55 ± 0.02	13.2 ± 0.3
> 2 and ≤ 4	2.53 ± 0.02	12.8 ± 0.4
> 4	2.60 ± 0.03	13.9 ± 0.4
β ± SE	0.06 ± 0.04Δ	0.07 ± 0.05Δ
P for trend	0.17	0.12
*BMI (kg/m^2^)*		
≤ 21.5	2.38 ± 0.02	11.2 ± 0.2
> 21.5 and ≤ 23.9	2.41 ± 0.02	11.5 ± 0.2
> 23.9	2.43 ± 0.02	11.7 ± 0.2
Std β ± SE	0.04 ± 0.04	0.04 ± 0.04
P for trend	0.22	0.27
*Active Intensity Index (METs)†*		
≤ 0.61	2.42 ± 0.02	11.6 ± 0.2
> 0.61 and ≤ 0.73	2.40 ± 0.02	11.5 ± 0.2
> 0.73	2.41 ± 0.02	11.5 ± 0.2
Std β ± SE	-0.02 ± 0.04	-0.03 ± 0.04
P for trend	0.56	0.45

* Values are age and sex adjusted Least Square Mean (LSM) ± Standard Error (SE) of natural logarithm of plasma total homocysteine concentration (log µmol/l).

† Log-transformed values were used for the multivariate linear regression analyses and the classification was based on tertiles.

‡ Values are age and sex adjusted least square mean (LSM) ± standard error (SE) of plasma total homocysteine concentration (µmol/l). Std β ± SE: Standardized regression coefficient ± standard error by a multivariate linear regression model of plasma tHcy concentration on each independent variable including sex and age as covariates.

¶ Caculated for 10 cigarettes.

Δ Caculated for 2 drinks.

### Associations between plasma tHcy and life-style factors according to MTHFR genotypes

As shown in Table 4, an inverse association between plasma tHcy and serum folate was stronger in the order of VV (std β = -0.42), AV (std β = -0.33), and AA (std β = -0.17) genotype, where the difference in the strength of association, expressed as std β, was statistically significant between VV and AV or AA genotype. Partial R^2^ implied that the inter-individual variance of plasma tHcy was more explained by the level of serum folate (partial R^2^ =0.075) than by MTHFR genotypes (partial R^2^ =0.011). Similarly, an inverse association between plasma tHcy and serum vitamin B_12_ was a little stronger in VV (std β=-0.29) than in AV (std β=-0.15) or in AA (std β=-0.11), but the difference of std β was not statistically significant among three genotypes. No association was observed between plasma tHcy and serum vitamin B_6_ in any genotype.

**Table 4.**
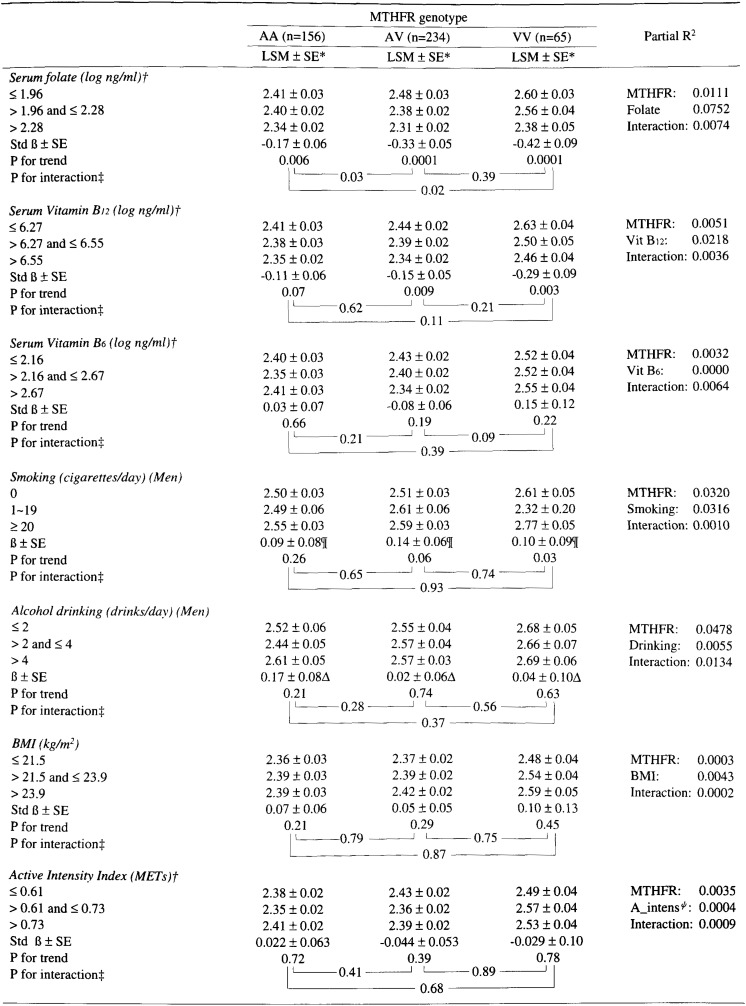
The effects of combinations between MTHFR genotypes and selected life-style factors onto the plasma total homocysteine concentration.

* Values are age and sex adjusted least square mean (LSM) ± standard error (SE) of natural logarithm of plasma tHcy concentration (log µmol/l).

† Log-transformed values were used for the multivariate linear regression analyses and the classification was based on tertiles.

Std β ± SE: Standardized regression coefficient ± standard error by a multivariate linear regression model of plasma tHcy concentration on each independent variable including sex and age as covariates.

‡ P for homogeneity of regression coefficient between genotypes.

¶ Caculated for 10 cigarettes.

Δ Caculated for 2 drinks.

*^ψ^* Active Intensity Index.

Plasma tHcy was higher in smokers, particularly in those with 20+ cigarettes/day than in non-smokers, but this was statistically significant only in VV genotype. Alcohol drinking, BMI, and physical activity were not related to plasma tHcy in any MTHFR genotype.

### Multivariate analysis of association of plasma tHcy with MTHFR genotype and selected life-style factors

As shown in Table 5, MTHFR VV genotype, serum folate, and serum vitamin B_12_ were independently associated with plasma tHcy. The interaction of serum folate × VV genotype also indicated that serum folate was more strongly associated with plasma tHcy in VV genotype than in AA genotype even in the multivariate analysis. Partial R^2^ of serum folate (0.0652) and that of serum vitamin B_12_ (0.0165) were larger than that of MTHFR genotype (0.0092). We added smoking habits as an independent variable to this model at the first step, but the association between smoking and tHcy was not significant (P=0.18) and, therefore, we did not include smoking in the final analysis shown in Table 5.

**Table 5.** Association of plasma tHcy with MTHFR genotypes and selected life-style factors in a multiple regression analysis.

	Std β ± SE	P	Partial R^2^	
Serum folate	-0.19 ± 0.09	0.003	MTHFR:	0.0092
MTHFR AV†	-0.006 ± 0.08	0.94	Folate:	0.0652
MTHFR VV†	0.41 ± 0.12	0.0004	Interaction:	0.0057
Serum folate × AV†	-0.14 ± 0.08	0.08‡	Vit B_12_:	0.0165
Serum folate × VV†	-0.24 ± 0.12	0.04‡		
Serum Vitamin B_12_	-0.14 ± 0.04	0.0002		

Std β ± SE: Standardized regression coefficient ± Standard Error by a multivariate linear regression model of plasma tHcy concentration on each independent variable after age and sex adjusted.

† Using AA as reference.

‡ P for homogeneity of regression coefficient between genotypes.

## DISCUSSION

Higher plasma tHcy is known as one of the strong risk factors for coronary heart disease and atherothrombotic stroke. A meta-analysis of 27 studies which investigated the relation between fasting tHcy levels and coronary artery disease (CAD) yielded an odds ratio of 1.6 for men and 1.8 for women for every 5 µmol/l increase in plasma tHcy level, and also showed that a 5 µmol/l increase in plasma tHcy elevated CAD risk by as much as an increase in total cholesterol of 20 mg/dl^18^^)^. A more recent review article indicated that the relative risk associated with a 5 µmol/l increment in the tHcy level was 1.7 (95% CI, 1.5-1.9) for coronary heart disease, 1.9 (95% CI, 1.6-2.3) for cerebrovascular disease, and even higher for peripheral artery disease^19^^)^. Most of these epidemiological studies demonstrated that such risks were independent of other known risk factors. In our study, there were no significant associations between plasma tHcy and other major traditional cardiovascular risk factors such as SBP, DBP, TC, HDL-C, body fat percentage (data not shown), and BMI. This suggested that elevated plasma tHcy could be an independent risk factor for CAD in this Japanese rural population, although we did not examine the relationship between tHcy and risks of CAD. Therefore, it would be important to conduct more cross-sectional studies like ours to gain the knowledge that would allow a more advanced discussion to develop control measures against elevated plasma tHcy.

Inherited disorders (MTHFR thermolability), which are present in approximately 5% of a general population^20^^)^, can alter enzyme activity in the transsulfurration and remethylation pathways^1^^,^^7^^,^^19^^,^^21^^,^^22^^)^, and these may result in elevated tHcy levels. The C677T MTHFR gene was shown to be responsible for the thermolabile enzyme^17^^,^^21^^,^^22^^,^^23^^)^ and the mutation altered the enzyme activity in the transsulfurration and remethylation pathways^1^^,^^7^^,^^19^^,^^21^^,^^22^^)^. In our study, the C677T MTHFR genotype mutation, particularly homozygous, was significantly associated with the elevated plasma tHcy. This finding was almost consistent with previous Japanese studies conducted with a relatively small number of individuals^17^^,^^22^^)^ or CAD patients^8^^)^. In addition, Frosst et al.^17^^)^ reported that the VV genotype resulted in reducing the activity of MTHFR enzyme and raising the level of plasma tHcy in French Canadian subjects. Several Japanese studies^9^^,^^10^^,^^11^^)^ showed that MTHFR mutation was significantly associated with the risk of CAD although such an association was not observed in several studies conducted in the US and Australian populations^24^^-^^26^^)^.

A multivariate linear regression analysis revealed that there was a significant inverse association between plasma tHcy and serum folate levels in individuals with any MTHFR genotype. Interestingly, we found that this inverse association was significantly stronger in VV genotype than in AA genotype. This finding supported the hypothesis that folate might stabilize the MTHFR enzyme activity^17^^)^; furthermore it may be consistent with the observation in which the oral supplementation of folic acid normalized hyperhomocysteinemia due to thermolabile MTHFR^27^^,^^28^^)^. Our results also supported that when folate concentration was high, this common genetic variant had little effect on tHcy levels^7^^,^^24^^,^^29^^,^^30^^)^. The present study demonstrated significant associations of MTHFR VV genotype with not only higher tHcy levels but also lower serum folate levels. This finding was consistent with the NHLBI Family Heart Study^7^^)^ and other studies^24^^,^^31^^)^. MTHFR catalyzes the synthesis of 5-methyltetrahydrofolate, main circulatory form of folate and the methyl donor in the remethylation of homocysteine to methionine^24^^,^^32^^,^^33^^)^. The VV genotype is associated with a thermolabile enzyme with 50% of normal activity^17^^)^. Our results suggest that the remethylation pathway becomes detectably inadequate, resulting in excess homocysteine, in the absence of sufficient substrate and the presence of suboptimal enzymatic activity.

Several studies demonstrated an inverse relationship between vitamin B_12_ status and plasma homocysteine levels^34^^-^^38^^)^ although the strength of the correlation was not as strong as that observed between folate status and homocysteine levels. Our result also showed that plasma tHcy was significantly and inversely associated with serum vitamin B_12_ concentration. Vitamin B_12_ in the form of methycobalamine is required as cofactor and folate in the form of 5-methyltetrahydrofolate is a co-substrate for the vitamin B_12_-dependent remethylation of homocysteine to methionine^23^^,^^39^^)^. Our result suggested that vitamin B_12_ was an essential factor as well as folate for the metabolism of homocysteine.

We did not find any relationship between serum vitamin B_6_ and plasma tHcy levels. Some previous studies reported a non-significant relationship^40^^-^^44^^)^ like ours whereas others reported a significant inverse relationship^36^^,^^45^^,^^46^^)^ between homocysteine levels and vitamin B_6_ status. Thus, currently the relationship between vitamin B_6_ and plasma homocysteine remained unclear. Further studies would be needed using larger samples controlling for potential confounders.

Smoking was significantly and positively associated with the plasma tHcy level. This result was very similar to other studies^10^^,^^31^^,^^47^^,^^48^^)^. Plasma tHcy level was positively associated with status of smoking in any genotype being significant only in VV genotype. In the multivariate analysis after adjusted for serum folate and vitamin B_12_, the relationship between smoking and plasma tHcy became non-significant. In addition, we also found that smoking was inversely associated with serum folate (r= -0.32, P=0.0001) and vitamin B_12_ (r= -0.20, P=0.0039) after adjusted for age and sex. Thus, it is plausible that cigarette smoking have caused a lower folate and vitamin B_12_ status and an eventual elevation of homocysteine concentration^49^^)^. Regardless of the individual’s MTHFR genotype, we should educate people to quit smoking for the prevention of hyperhomocysteinemia as well as other known many health problems.

Alcohol drinking was not associated with the level of plasma tHcy. Some studies observed that there was a modest association between alcohol intake and fasting tHcy, and significant positive associations were seen between tHcy and consumption of hard liquor and red wine, but not of beer^50^^,^^51^^)^. In our study, the majority of alcohol beverages consumed was beer. Therefore, the lack of association between alcohol drinking and plasma tHcy might be due to the type of alcohol beverages consumed in this population.

BMI was not associated with plasma tHcy and MTHFR genotype. This result supported to Gudnason et al^22^^)^, although Wilcken et al^25^^)^ observed that BMI was significantly associated with MTHFR mutation. BMI may be related to intake of total energy and hence other nutrients such as folate, vitamin B_12_, and B_6_. However, adjustment for BMI virtually unchanged the associations of folate, vitamin B_12_, and B_6_ with plasma tHcy (data not shown). Unlike some other studies^5^^,^^31^^,^^47^^,^^48^^)^, there was no association between plasma tHcy and daily physical activity in our study. We further need to study for elucidating other potential confounding factors to explain this result.

In the multivariate analysis, MTHFR mutant genotypes, serum folate, and serum vitamin B_12_ were independently associated with plasma tHcy levels. Although homocysteine level was independently influenced by both genetic (MTHFR) and environmental (folate and vitamin B_12_) factors, the partial R^2^ implied that the effects of folate and vitamin B_12_ on the tHcy level were larger than that of MTHFR gene. Furthermore, there was a significant gene-environmental interaction between MTHFR gene and serum folate. Therefore, future studies on the relationship between MTHFR gene and tHcy should be necessarily done considering such environmental factors.

Since the measurement of homocysteine is currently expensive, a mass screening for mild hyperhomocysteinemia may be impracticable. However, it would be a valuable advice for any people to take foods rich in folate (e.g., green soybeans, leafy and dark green vegetables, citrus fruits, citrus juice, and legumes) and vitamin B_12_ (e.g., seaweed, shell-fish, fin-fish, and poultry)^52^^,^^53^^,^^54^^)^, and to quit smoking.
